# Short rotator tendons do not increase intracapsular pressure in severe osteoarthritic hips

**DOI:** 10.1186/1471-2474-10-12

**Published:** 2009-01-25

**Authors:** Sarunas Tarasevicius, Arunas Gelmanas, Alfredas Smailys, Otto Robertsson, Hans Wingstrand

**Affiliations:** 1 Department of Orthopedics, Lund University Hospital, Getingevagen 4, Lund 22185 Sweden; 2 Departments of Anesthesiology, Kaunas Medical University, Eiveniu 2, Kaunas, LT-50009, Lithuania; 3 Departments of Orthopedics, Kaunas Medical University, Eiveniu 2, Kaunas, LT-50009, Lithuania

## Abstract

**Background:**

While a relation between pain and intracapsular pressure in the hip joint has previously been reported by some of the present authors, a newly published study including patients with severe osteoarthritis was not able to confirm this finding. This stimulated us to investigate the role of short rotators in relation to intracapsular pressure and pain in osteoarthritic hips.

**Methods:**

We measured the intracapsular hydrostatic pressure peroperatively in 25 total hip arthroplasty patients with severe osteoarthritis in various positions of the hip joint before and after short rotator release, and correlated these pressures to pain.

**Results:**

Release of the short rotators did not change the intracapsular pressure in any position except in 45° flexion, in which the pressure increased (p = 0.002). We found no correlation between intracapsular pressure and pain before or after short rotator release.

**Conclusion:**

We could not show that the rotators directly affected the pressure nor could we find a relation between pressure and pain.

## Background

It is generally accepted that an effusion in the hip joint is painful. Robertsson et al. [1] reported a correlation between intracapsular/hydrostatic pressure (ICP) and pain in osteoarthritis (OA). Goddard and Gosling [2] investigated OA patients and found that younger patients with more mobile hips had a higher resting ICP and more pain than older patients with less mobile hips. Some of the present authors [3] found that radiographic severity of OA was correlated to decreased elasticity/compliance of the joint capsule and to decreased intracapsular pressure. However, in that study the findings of Robertsson et al. [1], and Goddard and Gosling [2] of correlation of pressure and pain could not be confirmed. This might have been due to differences in ICP measurement technique [3]. Contrary to Robertsson et al. [3] we measured ICP in total hip replacements (THR) after the short rotators were released. Thus, our hypothesis was that release of short rotators might decompress the hip joint capsule and subsequently result in lower ICP and thus also explain the lack of correlation with pain observed in the previous study [3]. This would be in accordance with Lloyd-Roberts [4] and Goddard and Gosling [2] who suggested that pain in the OA hip may be due to the spasm and pressure of surrounding muscles on the hip joint capsule which is richly innervated [5,6]. Still, this hypothesis explaining such a pressure/spasm effect of the short rotator tendons on ICP has to our knowledge never been investigated or otherwise described in the literature.

The aim of our study was to investigate if perioperative release of the short rotator tendons in osteoarthritic hips affects ICP, and if there is a correlation between ICP and pain either before or after release of the short rotators.

## Methods

We analyzed 25 unselected OA patients admitted for elective THR. The radiographic grade of severity of OA was assessed according to Burnett et al. [7]. The evaluation is based on a number of radiographical criteria such as narrowing of the joint space, formation of osteophytes and cysts, and is graded in stages from 1 to11; the higher the grade the more severe radiographical OA.

A subscale of the HOOS [8] questionnaire evaluating hip pain was used. HOOS consists of 5 subscales; Pain, other Symptoms, Activities in daily living (ADL), Function in sport and recreation (Sport/Rec) and hip related Quality of life (QOL). The last week is taken into consideration when answering the questions. Standardized answer options are given (5 Likert boxes) and each question gets a score from 0 to 4. A normalized score (100 indicating no symptoms and 0 indicating extreme symptoms) is calculated for each subscale. The result can be plotted as an outcome profile.

Intracapsular pressure was measured perioperatively with a closed, non-volume consuming, sterile, monitoring set for invasive pressure (Braun, Combidyn Monitoring Set, Sensonor 840) connected to a pressure transducer. Care was taken to ensure that there were no air bubbles in the saline-filled tubes/epidural 18 gauge Tuohy needle. The system was zeroed and no fluid was introduced into the joint through the tubing system. With the hip joint exposed but the tendons of the short rotators intact, the joint capsule was penetrated with the 18 G needle. After penetrating the joint capsule, the needle was advanced onto the mid part of the femoral neck, twisted, and retracted about 1 mm to secure a free, intracapsular position of the tip. The hydrostatic intracapsular pressure with the hip in 45° of flexion was recorded, as well as in extension, extension-inward, and extension-outward rotation. Following these measurements with the needle still in the hip joint the tendons of short rotators were cut and measurements of hydrostatic intracapsular pressure were repeated in the same manner and in the same positions as with intact short rotators.

The paired t-test and Wilcoxon test were used to compare ICP in the hip joint in different positions before and after release of the short rotators. The p-values were the same independent of which method was used. The Spearman correlation was used to calculate the correlation between variables with a p value ≤ 0.05 considered to be significant. SPSS software was used for the calculations.

The study was approved by the ethical committee of the Kaunas Medical University Clinics.

## Results

The mean OA grade was 7.8 (SD 2.0), and the mean preoperative HOOS hip score for pain was 33.9 (SD 15.6). Before release of the rotators, the ICP in 45° flexion was significantly lower than in extension (Table 1). After release of the rotators the differences in pressure remained similar i.e. when comparing 45° of flexion to that in extension. However, when comparing differences before and after release we found that the only significant difference was that pressure increased in flexion after release (p = 0.002). In extension, outwards and inwards rotation of the hip the ICP remained almost unchanged (p > 0.5 for all positions).

**Table 1:** Intracapsular pressure measurements data with standard deviation

**Intracapsular pressure (mmHg)**	**45° flexion**	**Extension**	**Extension-inward rotation**	**Extension-outward rotation**
**Short rotators intact**	20.2 SD (17.0)	56.6 SD (82.5)	58.7 SD (77.3)	60.6 SD (89)
**Short rotators cut**	35.6 SD (29.5)	56.4 SD (67.3)	65.9 SD (78.6)	63.7 SD (74.1)

**Figure 1:**
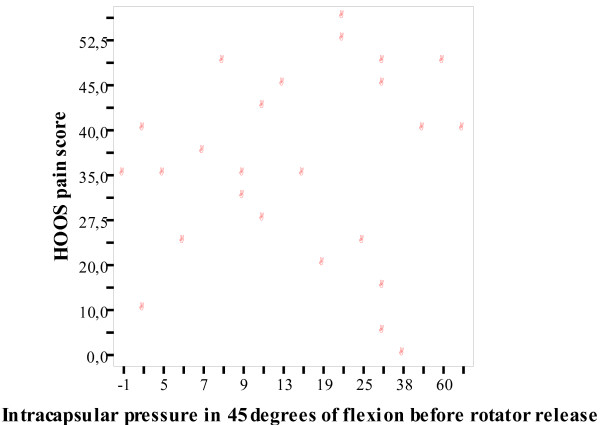
**Correlation between pain and intracapsular pressure in 45 degrees of flexion before short rotator release**. Correlation between pain and intracapsular pressure in 45 degrees of flexion.

**Figure 2:**
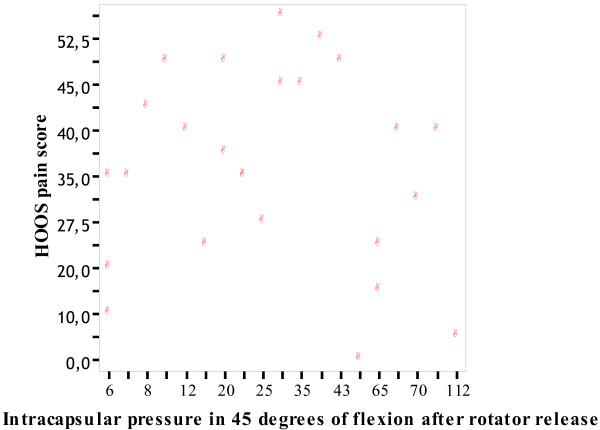
**Correlation between pain and intracapsular pressure in 45 degrees of flexion after short rotator release**. Correlation between pain and intracapsular pressure in 45 degrees of flexion.

We found no correlation between preoperative pain and ICP independent of position of the hip or release of the rotators or not (Table 2). Further, no correlation was found between OA grade and pain (Table 2). A graphical representation of the correlation between pain and intracapsular pressure in 45 degrees of flexion before and after short rotator release is presented in Figures 1 and 2.

**Table 2:** Spearman correlation between pain and intracapsular pressure before and after short rotator release and OA grade

**Variables**	**Pain**	
**Intracapsular pressure**	**Spearman correlation coefficient**	**p**

**Before tendons release in 45° of flexion**	0,13	0,5
**Before tendons release in extension**	0.08	0,7
**Before tendons release in outward rotation**	0,15	0,5
**Before tendons release in inward rotation**	0,15	0,5
**After tendons release in 45° of flexion**	-0,04	0,8
**After tendons release in extension**	-0,09	0,7
**After tendons release in outward rotation**	-0,02	0,9
**After tendons release in inward rotation**	-0,06	0,8
**OA grade**	-0,12	0,5

## Discussion

We did not find any change in intracapsular pressure (ICP) after release of the short rotators in radiographically severe OA hips in any position except in 45° of flexion in which the pressure increased after release. Thus, the short rotator tendons did not seem to generate any significant load on the hip joint capsule, or at least that this load was not sufficient to change the ICP in these hips. This is not in accordance with the hypothesis raised by previous authors [2,4] that surrounding hip joint muscles compress the hip joint capsule, subsequently causing pain. However, in the present study the majority of the patients had severe OA which has been reported to be accompanied by capsular contracture and decreased elasticity [3]. Thus it is possible that the capsules were so fibrotic and resistant to the forces generated by the rotators that ICP was not affected. Thus, in less severe OA, with more elastic capsules, a release of the short rotator tendons would have caused a change in ICP.

All pressure measurements were performed in patients under spinal anesthesia which might affect the degree of muscle relaxation and thus the intraarticular pressure. All previous studies [1-3] were conducted under the same anesthetic conditions as current one, thus results of the measurements of the pressures are comparable. However the effect of muscle relaxation is not estimated and might have some effect on the intraarticular pressure of the hip.

We found that release of the short rotators increased the ICP in 45° of flexion. We might speculate that the tendons in the arthritic hip are adherent to the capsule. Due to anatomy, in 45° of flexion, contraction exerts a pull on the capsule, decreasing the ICP. Release of the short rotators would consequently increase ICP.

We did not find any correlation between preoperative pain and ICP which also is not in accordance with some previous studies. Goddard and Gosling [2] found a correlation between ICP and pain. Robertsson et al. [1] who studied less radiographically severe OA cases (personal communication) as compared to our series found a correlation between higher ICP and more painful hips.

The levels of ICP in our study were similar to that reported by Robertsson et al. [1] but we found no correlation with pain. These findings support the theory of Goddard and Gosling [2] that OA in the hip may present itself as either a higher pressure in younger and more mobile hips or a lower pressure in older and less mobile hips. This implies that with time the OA "burns out" leaving the patient with less pain and motion.

It has been shown previously that a healthy capsule is very elastic and it can be stretched up to 55% in length without any plastic deformation [9]. There is very little joint fluid and no change in ICP within the normal range of motion in a healthy hip [9,10]. In the early development of OA, hyper production of joint fluid due to the synovitis may occur. In this early stage of OA the joint capsule probably is still elastic and can be stretched, increasing the intracapsular volume as has been observed sonographically in painful OA hips [11]. Such stretching could induce pain in early OA hips, correlating to higher intracapsular pressure in less severe disease [1] and younger and more mobile hips [2].

It has been observed that radiographically more severe OA is associated with a less elastic hip joint capsule [3] i.e. as OA progresses the hip joint capsule becomes less elastic. If pain is partially caused by stretching of the capsule, a fibrous, stiff capsule would be less sensitive to pressure and thus explain our lack of correlation between pressure and pain and would further suggest that a part of the pain in OA hips is of capsular origin.

## Conclusion

We conclude that the tension in the short rotator tendons does not increase the intracapsular pressure in radiographically severe OA hips, and that there is no correlation between pain and intracapsular pressure in late OA stages.

## Competing interests

The authors declare that they have no competing interests.

## Authors' contributions

ST data collection, compilation and analysis, writing manuscript. AG, AS data collection, editing manuscript. OT statistical analysis, editing manuscript. HW organizing study, editing manuscript. All authors read and approved the final manuscript.

## Pre-publication history

The pre-publication history for this paper can be accessed here:


